# Anticipatory plastic response of the cellular immune system in the face of future injury: chronic high perceived predation risk induces lymphocytosis in a cichlid fish

**DOI:** 10.1007/s00442-020-04781-y

**Published:** 2020-10-23

**Authors:** Denis Meuthen, Ingo Meuthen, Theo C. M. Bakker, Timo Thünken

**Affiliations:** 1 Evolutionary Biology, Bielefeld University, Konsequenz 45, 33615 Bielefeld, Germany; 2 Institute for Evolutionary Biology and Ecology, University of Bonn, An der Immenburg 1, 53121 Bonn, Germany; 3 Practice of Internal Medicine, Hematology and Oncology, Hauptstraße 39-41, 50996 Cologne, Germany

**Keywords:** *Pelvicachromis taeniatus*, Hematology, Lymphocytes, Phenotypic plasticity, Alarm cues

## Abstract

Vertebrate cellular immunity displays substantial variation among taxa and environments. Hematological parameters such as white blood-cell counts have emerged as a valuable tool to understand this variation by assessing the immunological status of individuals. These tools have long revealed that vertebrate cellular immune systems are highly plastic and respond to injury and infection. However, cellular immune systems may also be able to anticipate a high risk of injury from environmental cues (e.g., predation-related cues) and respond plastically ahead of time. We studied white blood-cell (leukocyte) profiles in African cichlids *Pelvicachromis taeniatus* that were raised for 4 years under different levels of perceived predation risk. In a split-clutch design, we raised fish from hatching onwards under chronic exposure to either conspecific alarm cues (communicating high predation risk) or a distilled water control treatment. Differential blood analysis revealed that alarm cue-exposed fish had twice as many lymphocytes in peripheral blood as did controls, a condition called lymphocytosis. The presence of a higher number of lymphocytes makes the cellular immune response more potent, which accelerates the removal of invading foreign antigens from the bloodstream, and, therefore, may be putatively beneficial in the face of injury. This observed lymphocytosis after long-term exposure to conspecific alarm cues constitutes first evidence for an anticipatory and adaptive plastic response of the cellular immune system to future immunological challenges.

**Electronic supplementary material:**

The online version of this article (10.1007/s00442-020-04781-y) contains supplementary material, which is available to authorized users.

## Introduction

To protect themselves against pathogens, the vertebrate immune system has evolved highly effective cellular immunity, of which white blood cells, also called leukocytes, are an important component. There are different types of leukocytes, ranging from cells with phagocytotic activity (neutrophils) to those that produce proteins such as antibodies (specialized lymphocytes called B cells). Hence, both the absolute amount and the relative frequency of different leukocytes characterize the cellular immune system response. Therefore, hematology, the study of blood, was developed since the 1920s as a valuable and highly informative medical diagnostic tool (Wintrobe et al. 1974). Researchers have since used differential leukocyte counts for studying variation in wildlife immune responses (Davis et al. 2008), but this variation is still not fully understood (Maceda-Veiga et al. 2015). Most previous studies have been conducted in a medical, toxicological, and animal ethics context, and thus focus on the consequences of exposure to environmental factors that disturb physical integrity such as toxins (Eeva et al. 2005; Villa et al. 2017), parasites, and pathogens (Davis et al. 2004; Lobato et al. 2005; Burnham et al. 2006), suboptimal nutrition, temperature, or humidity levels (Bennett and Daigle 1983; Altan et al. 2000; Brown and Shine 2018; Włodarczyk et al. 2018; Roast et al. 2019), as well as animal handling (Morrow-Tesch et al. 1993; Dhabhar et al. 1994; Kock et al. 1999; Lance and Elsey 1999; Zapata et al. 2004; Davis and Maerz 2011). Only a few studies have investigated variation in differential leukocyte counts from other perspectives, such as phylogenetic comparisons between species (Minias et al. 2018; Downs et al. 2020) or ontogeny-related variation in cellular immunity (Dehnhard et al. 2011; Jakubas et al. 2015).

However, to our knowledge, no previous study has considered that vertebrate cellular immune systems may also respond adaptively to non-integrity-disturbing cues that are indicative of an environment with increased injury risk. In the face of possible future injury, a cue-induced proliferation of cellular immune system components has the potential to fight off pathogens early and thereby may vastly reduce disease-related fitness costs. This may constitute another case of how adaptive phenotypic plasticity allows individuals to adapt to changing environments (West-Eberhard 2003; Scheiner et al. 2020), similar to how prey animals respond plastically to the key ecological factor predation (Lima and Dill 1990; Nosil and Crespi 2006). During antipredator phenotypic plasticity, cues that communicate high predation risk induce plastic modifications in the behavior, morphology, and life-history of prey animals, which increases individual fitness in a predatory habitat (Ghalambor et al. 2007; Kishida et al. 2010; Bourdeau and Johansson 2012). As predation is an environmental factor that substantially increases injury risk in any given environment (e.g., Reimchen 1988), it also provides a well-suited context for research on the adaptive plasticity of cellular immune systems. In vertebrates, antipredator phenotypic plasticity has first been discovered in a fish species, the crucian carp *Carassius carassius*. In this species, exposure to predators (Brönmark and Miner 1992) or to conspecific alarm cues (Stabell and Lwin 1997) triggers the development of a deeper body morphology (i.e., increased dorsoventral height) that decreases the risk of being swallowed by gape-limited piscivores such as the pike *Esox lucius* (Nilsson et al. 1995). Similar patterns of morphological antipredator plasticity have since then been confirmed in many other fish species (Eklöv and Jonsson 2007; Januszkiewicz and Robinson 2007; Frommen et al. 2011; Meuthen et al. 2018a, 2019e). While there is also a lot of evidence for behavioral (Ferrari et al. 2015; Kim 2016; Meuthen et al. 2019d, 2019c) and life-history antipredator phenotypic plasticity (Reznick and Endler 1982; Belk 1998; Johnson and Belk 2001; Dzikowski et al. 2004) across fish taxa, no single study has considered that the fish cellular immune system may likewise respond with adaptive plasticity to perceived predation risk.

Fish hematology has a long history (Hesser 1960; Blaxhall and Daisley 1973), and this is why, fish are a well-studied, non-human vertebrate group in terms of their leukocyte responses (Davis et al. 2008; Burgos-Aceves et al. 2019). Ichthyologists consider fish leukocyte responses one of the most sensitive indicators of stress (Wedemeyer et al. 1990). Hence, many researchers have studied changes in fish leukocyte frequencies following exposure to stressors. Some of these researchers suggest that exposure to stress increases neutrophil numbers (neutrophilia) and decreases lymphocyte counts (lymphopenia), which leads to an elevated neutrophil:lymphocyte ratio (Larsson et al. 1980; Pulsford et al. 1994; Witeska 2005; Campbell 2012; Grzelak et al. 2017). In contrast, other studies report that exposure to stressful environmental factors induces an increase in lymphocyte frequency (lymphocytosis) and a decrease in neutrophils (neutropenia) (Johansson-Sjöbeck and Larsson 1978; Nussey et al. 1995). Although they had diverging results, these studies were similar in that they performed acute exposure to environmental factors that disturb individual physical integrity. Even when there is mention of a chronic exposure protocol, this refers to a period of no more than up to 9 weeks and a 9-week exposure period was only applied in a single study (Johansson-Sjöbeck and Larsson 1978). However, because fish are ectothermic, the time course of fish leukocyte patterns is lengthy (Davis et al. 2008), and hence, they reflect long-term stress more accurately than short-term stress as directly shown in a study with the channel catfish *Ictalurus punctatus* (Bly et al. 1990). Hence, there is a clear need for more long-term research to understand patterns of phenotypic plasticity in fish leukocytes.

Here, we study differential leukocyte profiles in response to long-term perceived predation risk in the Western African cichlid *Pelvicachromis taeniatus* (Lamboj 2004), also known as *P. kribensis* (Lamboj 2014)*.* This socially monogamous, stream-dwelling fish with complex mutual mate choice (Thünken et al. 2012) and biparental care (Thünken et al. 2010) is a prime example for antipredator phenotypic plasticity. In this species, predation risk is communicated through alarm cues that are detected by conspecifics (Meuthen et al. 2014, 2018b). Long-term exposure to high perceived predation risk as communicated through these cues during development plastically induces generalized neophobia (Meuthen et al. 2016). In adult fish, high perceived risk during development induces male-specific morphological modifications (Meuthen et al. 2018a), alters loser strategies during intrasexual competition (Meuthen et al. 2019a), and plastically adjusts mate preferences by lowering investment into mate choice (Meuthen et al. 2019b). Our aim here was to study the impact of the same developmental environment on the cellular immune system in the *P. taeniatus* individuals from the studies by Meuthen et al. (2016), Meuthen et al. (2018a), Meuthen et al. (2019b), and Meuthen et al. (2019a). To ensure that we studied antipredator plasticity in the differential leukocyte profiles of *P. taeniatus* rather than a short-term response to environmental modification, we investigated the immune response of *P. taeniatus* after individuals had completed more than half of their lifetime under high perceived predation risk. *P. taeniatus* reaches sexual maturity at 1–1.5 years age and can live up to 6 years in age (D. Meuthen, personal observation), and hence, we sampled fish at 4 years of age. At this time point, we obtained blood samples from *P. taeniatus* that had been raised under continuous exposure to either alarm cues or a water control treatment. With these samples, we then prepared stained peripheral blood smears and obtained differential leukocyte counts with light microscopy. Lymphocytes, the immune cells that have cytotoxic capabilities and produce antibodies (Campbell 1996), are the most common leukocytes in fish (Campbell 2012). Because they play a crucial role in host defense against pathogens (e.g., Rouse and Babiuk 1975; Gautreaux et al. 1994), an increased lymphocyte frequency (lymphocytosis) is a common response to infections across vertebrates and in fish also occurs in response to a high-quality diet (Fagbenro et al. 2013; Rashidian et al. 2020). The fact that vertebrates with immunodeficient mutations causing lymphopenia are particularly susceptible to infections (mice: Bosma and Carroll 1991; Rozengurt and Sanchez 1993; humans: Buckley et al. 1997; Villa et al. 2001) demonstrates the protective role of lymphocytes. Accordingly, a higher number of lymphocytes may accelerate the removal of pathogens from the bloodstream, and are therefore putatively beneficial in the face of injury.

However, increased lymphocyte production is not without costs—it requires a higher resource investment, and it is also likely to accumulate DNA replication errors, which may ultimately lead to cancerous growth (Stetler-Stevenson 2005; Vineis et al. 2010; Greaves and Maley 2012). Hence, only in individuals that inhabit an environment with elevated risk of injury, such as an environment with high perceived predation risk, lymphocytosis would constitute a putatively beneficial plastic response of the cellular immune system. Consequently, we predict a higher number of lymphocytes in alarm cue-exposed *P. taeniatus* as opposed to controls. Alternatively, as a typical stress response, we would expect lower lymphocyte and higher neutrophil numbers in peripheral blood, which causes an elevated neutrophil:lymphocyte ratio (Larsson et al. 1980; Pulsford et al. 1994; Witeska 2005; Campbell 2012; Grzelak et al. 2017). Because leukocyte patterns might be sex-dependent (Evans 2008) and previous research highlights the relevance of sex-specific plasticity in the study species (Meuthen et al. 2018a) and other fishes (Meuthen et al. 2019e), we also considered the sex of the experimental fish in our analyses.

## Materials and methods

### Rearing and treatment protocol

The fish used in the present experiment were derived from 60 wild-caught individuals collected in June 2007 from the Moliwe river in Cameroon (04°04′ N, 09°16′ E) that were afterwards bred in captivity. In 2012, adult F1 fish were paired up in different combinations so as to set up 12 outbred pairs, from which we derived the clutches used in the present study. After collecting the clutches, we split them into two equally sized groups and then exposed fry from hatching onwards for 5 days a week over 3 years to two different chemical cues that communicated different levels of perceived predation risk. First, to control for possible effects of frequent water disturbance, we applied a low-risk control treatment that consisted of exposure to distilled water. Second, we exposed the other half of each clutch to conspecific alarm cues derived from ground whole conspecifics (a combination of four male and four female donor fish in every instance) in a concentration of 7.2 mg/l as a proxy for high perceived predation risk; alarm cue preparation has been described in more detail in Meuthen et al. (2019b). The applied alarm cue concentration has previously been shown to induce behavioral (Meuthen et al. 2016, 2019a, b) and morphological (Meuthen et al. 2018a) antipredator phenotypic plasticity in *P. taeniatus* and in other fish species (Chivers and Smith 1994). The benefits of using conspecific alarm cues to generate high perceived predation risk are that fish do not habituate to them even after chronic exposure, while they do in response to predator odors (Imre et al. 2016). Furthermore, exposure to conspecific alarm cues is known to generate similar phenotypes as in fish from natural water bodies that house predators (Stabell and Lwin 1997; Laforsch et al. 2006; Meuthen et al. 2019d). Throughout rearing, fish were kept in mixed-sex groups of up to ten individuals per tank; we increased tank sizes sequentially to conform to the increased space requirements of growing fish (age 22–220 days: 20 × 30 × 20 cm, age 220–1664 days: 50 × 30 × 30 cm). Furthermore, we matched food amounts to fish number and ontogenetic stage as antipredator plasticity has been suggested to be limited by nutrient availability (Chivers et al. 2008); stated are the days from which onwards the given food amounts were supplied: 8–13 d: 10 µl/fish; 22-27d: 20 µl/fish; 50–55 days: 40 µl/fish; 78–83 days: 60 µl/fish; 115–122 days: 80 µl/fish; 150–157 days: 100 µl/fish; 185–192 days: 120 µl/fish; 220–227 days: 140 µl/fish; 255–262 days: 160 µl/fish; 297–304 days: 180 µl/fish; 339–346 days: 200 µl/fish. At first, food consisted of *Artemia* nauplii exclusively; from 115–122 days onwards it was replaced by a mix of frozen *Artemia* sp. and *Chironomus*, *Culex* as well as *Chaoborus* larvae in a ratio of 2:1:0.25:1. Throughout rearing, fish in different tanks had no visual or olfactory contact, water temperature was kept constant at 24.5 ± 1.5 °C, and illumination was provided by full-spectrum fluorescent tubes (Lumilux Cool Daylight 36 W/865, Osram, Germany) in a 12:12 light:dark cycle (from 8 am to 8 pm). In 2017, we derived 4-year old fish (age 1488–1664 days) from this split-clutch design to study variation in cellular immune system responses between treatments.

### Experimental procedure

To collect blood samples, we individually removed fish from their home tank and first assessed fish size (standard length: distance from the snout tip to the base of the tail fin) to the nearest millimeter with graph paper as well as fish body mass to the nearest milligram using a digital precision scale (LC221S, Sartorius, Göttingen, Germany). Afterwards, we immediately killed the fish by hypothermal shock as induced by immersion in ice slurry at 0–4 °C temperature to collect blood samples. *P. taeniatus* did not show any signs of distress during this procedure and hypothermal shock is a well-established method of euthanasia that is less stressful for small, tropical fish relative to benzocaine and MS-222 exposure (Wilson et al. 2009; Blessing et al. 2010; Lidster et al. 2017). Furthermore, exposure to MS-222 is known to modify blood properties and leukocyte histology (Palic et al. 2006; Popovic et al. 2012) and is, therefore, unsuitable for the study of leukocyte profiles. Blood samples were then collected by puncturing the heart from below the gill covers with a 10 µl syringe (Microliter 701, Hamilton, USA). A small drop of blood was then put on a standard microscope slide (soda-lime glass with frosted edge, H868, Carl Roth, Germany). Afterwards, we placed a second slide (edge ground at a 45° angle) at 40° degrees angle against the surface of the first slide and drew it back to contact the drop of blood which then spread over the interface of the slide through capillarity. Then, we quickly pushed the slide in the opposite direction, which created a blood smear. We did not use anticoagulants so as to prevent modification of the morphology of certain leukocytes, which makes their classification difficult (Ellis 1977). We always prepared several slides per individual fish, which were then labeled with fish identity codes. Blood smears were left to dry for at least 2 days. Afterwards, we conducted differential staining by May–Gruenwald–Giemsa (Pappenheim stain). The staining protocol consisted of first submerging slides for 3 min in an eosine methylene blue solution with at least 80% methanol for fixation (May–Gruenwald's solution, T863, Carl Roth, Germany). Then, slides were rinsed with distilled water and afterwards submerged in an azure, eosine, methanol, and glycerin solution (Giemsa stock solution diluted in a ratio of 1:20, T862, Carl Roth, Germany). Afterwards, slides were again rinsed with distilled water and then left to dry.

**Fig. 1:**
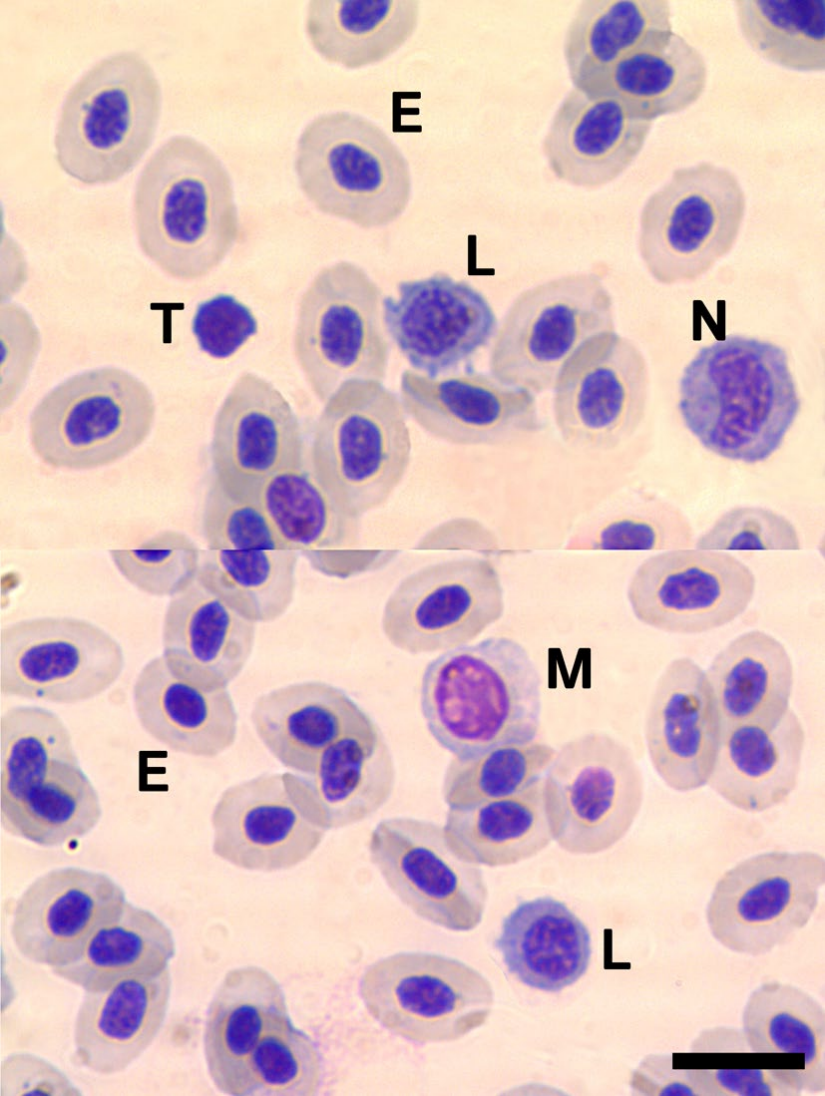
Photomicrographs (100 × magnification) displaying the morphology of the peripheral blood cells in *Pelvicachromis taeniatus*. Peripheral blood smears were stained by May–Grünwald–Giemsa (Pappenheim stain). *E* Erythrocyte, *L* Lymphocyte, *T* Thrombocyte, *N* Neutrophil, and *M* Monocyte. To allow a better comparison between different cell types, one lymphocyte (in the bottom image), the thrombocyte, and the neutrophil were copied from a photograph taken from a different area of the same blood smear at the same magnification and inserted into the above images with an image editor. The scale bar equals 10 µm

After all blood smears were stained and dried, the best slide (i.e., the slide that had the least signs of coagulation and the most intact cells) was selected for each individual, and blood smears were examined with an Axiolab light microscope (Carl Zeiss, Jena, Germany) at 400 × magnification by a hematologist (IM) that was naïve as to individual treatment. First, we conducted an initial qualitative differentiation of the different white blood cells in this species (Fig. 1). Afterwards, to quantify cellular immunity levels, for each slide, we first estimated absolute leukocyte counts at an accuracy of ± 50 leukocytes/µl. Then, thin areas of the blood smears where erythrocytes overlapped for a maximum of 1/3 of cell volume or alternatively, did not overlap at all, were examined for differential blood analysis. Here, we counted 100 randomly selected leukocytes per slide and assigned counts to their respective cell type. We followed a standard leukogram procedure by counting lymphocytes, neutrophils, eosinophils, basophils, monocytes, and erythroid/neutrophile precursors. As basophils, eosinophils, and precursors were very rare (found to be present in only 11.24%, 1.24%, and 0% of all blood smears, respectively and equally distributed across treatments), we excluded them from our analysis. From these relative values, absolute blood counts were then calculated for each individual fish as well as the proportion of neutrophils:lymphocytes as this ratio is suggested to be a reliable indicator of stress (Davis et al. 2008). Observed lymphocytes were polymorph (different cell sizes, different core sizes, different core-cytoplasm ratios, and different chromatin structures) throughout. In total, we collected blood from 44 alarm cue-exposed fish (21 females and 23 males) and from 45 control fish (27 females and 18 males). At the point of sampling, males from different treatments did not differ in body size (median, interquartile range, IQR; alarm cue-exposed fish: 8.3 cm, 8.1–8.6 cm; control fish: 8.2 cm, 8.0–8.7 cm; Wilcoxon signed-rank test: *W* = 225, *p* = 0.644) or weight (alarm cue-exposed fish: 7.182 g, 6.073–8.108 g; control fish: 6.937 g, 6.254–7.943 g; Wilcoxon signed-rank test: *W* = 214, p = 0.866). Likewise, females did not differ in body size (alarm cue-exposed fish: 5.8 cm, 5.7–6.0 cm; control fish: 5.9 cm, 5.7–6.0 cm; Wilcoxon signed-rank test: *W* = 253.5, *p* = 0.535) or weight (alarm cue-exposed fish: 2.876 g, 2.681–2.977 g; control fish: 2.897 g, 2.555–3.118 g; Wilcoxon signed-rank test: *W* = 275.5, *p* = 0.876) between treatments.

### Statistical analysis

For statistical analysis, we used R 3.2.5 (R Core Team 2016). After log-transformation, all variables met assumptions of normality according to Shapiro–Wilk tests (function “shapiro.test” in R package “stats”), and hence, we applied parametric tests throughout. We constructed linear mixed-effects models (function “lme” in R package “nlme”, Pinheiro et al. 2016) with maximum-likelihood parameter estimation throughout. Here, we always entered “fish family” as random intercept so as to account for genetic effects. All results are based on likelihood ratio tests (LRT), which assessed whether the removal of a variable caused a significant decrease in model fit according to the Aikake information criterion; hence, degrees of freedom differed by one in all models. The reported *P* values refer to the increase in deviance when the respective variable was removed.

To determine how leukocyte profiles differed between individuals, we constructed a model with the respective blood parameter (leukocytes, lymphocytes, neutrophils, monocytes, and proportion neutrophils:lymphocytes) as dependent variable and “sex” (male, female) as well as “treatment” (alarm cue-exposed, control) as explanatory variable. To determine whether sexes differed in their response to the treatment, we analyzed the “sex × treatment” interaction. When no significant interaction was present, we tested first for the general effects of sex, while treatment remained in the model as a covariate. Finally, when general sex effects were absent as well, we aimed to determine which blood parameter variation was affected by the treatment by testing treatment effects in the absence of any covariates. All initial and final models are available in the supplementary material (Online Resource 1).

## Results

Male and female leukocyte profiles did not differ in their response to the treatment (“interaction sex × treatment”, LRT: leukocytes, *χ*^2^ = 0.117, *p* = 0.732; lymphocytes, *χ*^2^ = 0.321, *p* = 0.571; neutrophils, *χ*^2^ = 0.006, *p* = 0.939; monocytes, *χ*^2^ = 2.585, *p* = 0.108; proportion neutrophils:lymphocytes, *χ*^2^ = 0.152, *p* = 0.697). In general, male and female blood parameters did not differ significantly (LRT; leukocytes, *χ*^2^ = 0.020, *p* = 0.888; lymphocytes, *χ*^2^ = 0.002, *p* = 0.965; neutrophils, *χ*^2^ = 0.022, *p* = 0.883; monocytes, *χ*^2^ = 0.725, *p* = 0.395; proportion neutrophils:lymphocytes, *χ*^2^ = 0.152, *p* = 0.697).

**Fig. 2:**
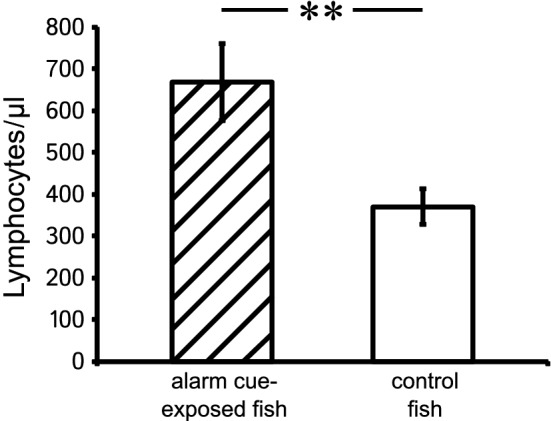
Absolute lymphocyte numbers (mean ± SE) in peripheral blood smears of 4-year old *P. taeniatus* that were subject to a lifelong difference in levels of perceived predation risk (alarm cue-exposed fish, dashed bar, *N* = 44; control fish, white bar, *N* = 45). **p = 0.002

However, we found significant treatment effects (Table 1). Fish from the alarm cue exposure treatment had approximately 30% more leukocytes (LRT, *χ*^2^ = 5.693, *p* = 0.017), which was caused by a doubling of lymphocyte counts in alarm cue-exposed individuals (LRT, *χ*^2^ = 9.512, *p* = 0.002, Fig. 2). In contrast, the other blood parameters did not differ significantly between treatments: neutrophils (LRT, *χ*^2^ = 2.767, *p* = 0.096); monocytes (LRT, *χ*^2^ = 1.997, *p* = 0.158); proportion neutrophils:lymphocytes (LRT, *χ*^2^ = 0.222, *p* = 0.638).

**Table 1:** Leukocyte profiles (mean ± SE) in peripheral blood smears of 4-year old. *P.* *taeniatus* that were lifelong subject to different levels of perceived predation risk: alarm cue-exposed fish (*N* = 44) and control fish (*N* = 45). All values are accompanied by the results of our final linear mixed-effect models that analyzed whether treatment explained variation in blood parameters, while fish family was included as a random intercept to account for our split-clutch design with multiple families

Cell type	Control-exposed	Alarm cue-exposed	*χ*^2^	*p*
Leukocytes	805.556 ± 125.652/µl	1278.409 ± 190.695/µl	5.693	0.017
Lymphocytes	370.233 ± 43.368/µl	668.375 ± 91.803/µl	9.512	0.002
Neutrophils	416.633 ± 93.622/µl	580.034 ± 115.672/µl	2.767	0.096
Monocytes	17.461 ± 4.259/µl	28.727 ± 6.758/µl	1.997	0.158
Proportion neutrophils:lymphocytes	1.038 ± 0.130: 1	0.891 ± 0.090: 1	0.222	0.638

## Discussion

Our results revealed that alarm cue-exposed fish had a significantly higher absolute number of leukocytes (i.e., total white blood cells) which was caused by a significantly greater number of lymphocytes in alarm cue-exposed *P. taeniatus* relative to the water control. Instead, we did not find evidence for changes in the frequency of other blood-cell types or in neutrophil:lymphocyte proportions. Given the crucial role of lymphocytes in the host defense against pathogens (e.g., Rouse and Babiuk 1975; Gautreaux et al. 1994), having a higher number of lymphocytes likely benefits vertebrates in the face of injury, which is more likely to occur in an environment with high predation risk (Reimchen 1988). Hence, this observed lymphocytosis is first evidence for putatively beneficial phenotypic plasticity in a vertebrate cellular immune system. More generally, it is also the first evidence for a preceding putatively beneficial immunological response in an environment with increased injury risk. While, in our study, we used non-integrity-disturbing cues that communicate high perceived predation risk, lymphocytosis has previously been observed as a response to dietary supplementation in the rainbow trout *Oncorhynchus mykiss* (Rashidian et al. 2020), to copper exposure in the Mozambique tilapia *Oreochromis mossambicus* (Nussey et al. 1995), and as a response to cadmium exposure in the flounder *Pleuronectes flesus* (Johansson-Sjöbeck and Larsson 1978). Likewise, in humans, chronic stress (Pereira et al. 2012), cigarette smoking (Chan et al. 1990; Tollerud et al. 1991; Delannoy et al. 1993; de Haan and Pouwels 2006), or chronic viral and bacterial infections (Speight et al. 1999; Halim and Ogbeide 2002; Sever-Prebilic et al. 2002; Chabot-Richards and George 2014) have all been suggested to induce lymphocytosis.

At first glance, our observation of an induced lymphocytosis in response to chronic exposure to high perceived predation risk appears contradictory to previous research. That is because similar to other stressors (Barcellos et al. 2011), perceived predation risk is suggested to induce an increase in the levels of the stress hormone cortisol (a glucocorticoid) as has previously been suggested in studies on fish transgenerational antipredator plasticity (Giesing et al. 2011; Sopinka et al. 2015). Elevated glucocorticoid levels then trigger a redistribution of leukocytes between body compartments (Davis et al. 2008): a rapid release of neutrophils from the head kidney into peripheral blood (which causes neutrophilia in the blood) and a mobilization of lymphocytes from circulating blood into compartments such as the skin, the spleen, and lymph nodes (which causes lymphopenia in the blood: Dhabhar et al. 1996; Dhabhar and McEwen 1997). This process then results in an elevated neutrophil:lymphocyte ratio in peripheral blood as has been shown multiple times as a consequence of exposing fish to other stressors (metals: Larsson et al. 1980; Witeska 2005; forced upside-down position: Pulsford et al. 1994; higher temperature and longer photoperiods: Campbell 2012; exposure to air: Grzelak et al. 2017). Despite potential short-term benefits of having more lymphocytes in specific body compartments as a preparation for injury (Johnstone et al. 2012), other researchers consider stress-induced lymphopenia in peripheral blood to be an immunosuppressive condition that impairs wound healing as showcased in mice (Padgett et al. 1998; Padgett and Glaser 2003).

However, cellular immune responses to glucocorticoid exposure are different when it comes to chronic stress where these hormones are released continuously. Under these conditions, glucocorticoid receptor levels are typically down-regulated (Svec and Rudis 1981; Vedeckis et al. 1989; Cohen et al. 2012) so as to avoid the negative effects on the vertebrate body that is associated with prolonged glucocorticoid exposure (Russell and Lightman 2019). Because lymphocytes also carry glucocorticoid receptors, lymphocyte sensitivity to glucocorticoid exposure decreases as well (Wodarz et al. 1991; Bauer et al. 2000). Likewise, neutrophil-secreted pro-inflammatory cytokines such as interleukin-8 are known to adjust the relative amounts of glucocorticoid receptors on other neutrophils so as to make them less sensitive to glucocorticoids, which avoids glucocorticoid-induced cell-death (Strickland et al. 2001). Hence, under chronic stress, despite continued glucocorticoid release, both lymphocyte and neutrophil numbers in peripheral blood are supposed to reach normal levels again, and this is likely the reason why we did not observe an elevated neutrophil:lymphocyte ratio as is typical for most studies on the consequences of acute stress. However, the effect of glucocorticoids on the vertebrate cellular immune system is now known to be more complex than anticipated; they have not only anti-inflammatory effects such as lymphopenia, but contradictorily can also have pro-inflammatory effects such as lymphocytosis, a phenomenon that researchers have only recently started to understand (Cruz-Topete and Cidlowski 2015). Additionally, lymphocyte frequencies are known to be more sensitive to glucocorticoid levels compared to neutrophils (Cole et al. 2009). Hence, the putatively beneficial lymphocytosis that we observed in our study may still have been triggered through chronic predator-related glucocorticoid releases.

On the other hand, the plasticity-mediated maintenance of a chronic lymphocytosis is not without potential costs. This is because as the probability of mutations increases with each cell replication event, a chronically increased production of lymphocytes is likely to accumulate DNA replication errors. Clonal selection and tumor progression models (Stetler-Stevenson 2005; Vineis et al. 2010; Greaves and Maley 2012) predict that such mutations then have the potential to cause a switch from a beneficial lymphocytosis to a malignant lymphocytosis such as, for example, a monoclonal B-cell lymphocytosis (MBA). In humans, MBA is an asymptomatic precursor condition for malignant chronic lymphatic leukemia (Shim et al. 2010; Mowery and Lanasa 2012). This theoretical tumor progression is confirmed by studies on humans, suggesting that persistent reactive polyclonal B-cell lymphocytosis can develop into malignant disorders such as lymphomas (de Haan and Pouwels 2006; Xochelli et al. 2015). As these malignant diseases are lethal, a shorter lifespan induced by the observed chronic lymphocytosis is likely to constitute one of the costs of cellular immune system plasticity that is outweighed only in environments with high injury risk. In line with the theory that traits only evolve to be plastic if they are costly (Ghalambor et al. 2007), this may be why an elevated proliferation of lymphocytes has evolved as a plastic rather than a fixed response.

Future studies are required to expand on our findings. Because of the low amount of blood that we could collect in our experimental fish (~ 0.5 to 5 µl per individual), we could not measure glucocorticoid concentrations as performing such an analysis requires approximately 30–60 µl of blood. Hence, it is important to set up studies that measure how vertebrate glucocorticoid concentrations change over time in an experiment with chronic (i.e., over 50% of an individuals’ lifetime) exposure to stress. Additionally, researchers should aim to reveal on a cellular level why chronic exposure to stress only impacts on lymphocyte but not neutrophil numbers or neutrophil:lymphocyte ratios. Furthermore, attempts should be made to directly determine the adaptive benefit of the observed lymphocytosis as induced by chronic exposure to an environment with high perceived predation risk. To do so, one would have to artificially injure fish that had previously been chronically exposed to the same treatments as here and afterwards statistically compare wound healing speed, probabilities to develop diseases, as well as mortality rates between treatments. Further follow-up studies should also aim to directly measure the costs associated with chronic lymphocytosis by comparing the probability of leukemia occurrence as well as maximum lifespan between fish from the same treatments. More generally, future research should also attempt to find additional examples for anticipatory plasticity of vertebrate cellular immune systems, and to do so, expand the hitherto lacking research on the consequences of chronic exposure to stressors that are associated with increased future injury probability. At the same time, immunological research should focus more on the impact of environmental cues that do not disturb physical integrity, which has been underrepresented to date.

## Electronic supplementary material

Below is the link to the electronic supplementary material.

Supplementary file1 (HTML 632 kb)
