# Supplementary material for: Anticipatory plastic response of the cellular immune system in the face of future injury: chronic high perceived predation risk induces lymphocytosis in a cichlid fish
Source: Oecologia. 2020 Oct 23;194(4):597–607. doi: 10.1007/s00442-020-04781-y (PMC7683483; doi:10.1007/s00442-020-04781-y)
Supplement: Supplementary file 1 — Supplementary file1 (HTML 632 kb) [file 442_2020_4781_MOESM1_ESM.html]

Initial and final models for paper: ‘Anticipatory plastic response of the cellular immune system in the face of future injury: Chronic high perceived predation risk induces lymphocytosis in a cichlid fish’


# Initial and final models for paper: "Anticipatory plastic response of the cellular immune system in the face of future injury: Chronic high perceived predation risk induces lymphocytosis in a cichlid fish"

## Oecologia

#### Denis Meuthen, Ingo Meuthen, Theo C. M. Bakker, Timo Thünken

##### Corresponding author: Denis Meuthen, denis.meuthen@uni-bielefeld.de, Evolutionary Biology, Bielefeld University, Konsequenz 45, D-33615 Bielefeld, Germany. Tel.: ++49 521 106 28 20. Fax: ++49 521 106 64 26

  

```
library(nlme)
```

```
Dataset <- read.table("clipboard", header=TRUE, sep="\t", na.strings="NA", dec=".", strip.white=TRUE)
```

## Leukocytes

### Initial model

```
lme.1 <- lme(log(Leuco)~Sex*Treatment, random=~1|Family, data=Dataset, method="ML", na.action="na.omit")
summary(lme.1)
```

```
## Linear mixed-effects model fit by maximum likelihood
##  Data: Dataset 
##        AIC     BIC    logLik
##   239.9732 254.905 -113.9866
## 
## Random effects:
##  Formula: ~1 | Family
##         (Intercept) Residual
## StdDev:   0.2031439  0.85176
## 
## Fixed effects: log(Leuco) ~ Sex * Treatment 
##                     Value Std.Error DF  t-value p-value
## (Intercept)      6.787905 0.2051851 73 33.08186  0.0000
## Sexm            -0.090010 0.2687084 73 -0.33497  0.7386
## TreatmentC      -0.512977 0.2595270 73 -1.97658  0.0519
## Sexm:TreatmentC  0.130001 0.3879486 73  0.33510  0.7385
##  Correlation: 
##                 (Intr) Sexm   TrtmnC
## Sexm            -0.674              
## TreatmentC      -0.713  0.552       
## Sexm:TreatmentC  0.477 -0.714 -0.688
## 
## Standardized Within-Group Residuals:
##         Min          Q1         Med          Q3         Max 
## -2.64434375 -0.65355675 -0.05732818  0.55884514  2.44510547 
## 
## Number of Observations: 89
## Number of Groups: 13
```

```
lme.2 <- lme(log(Leuco)~Sex+Treatment, random=~1|Family, data=Dataset, method="ML", na.action="na.omit")
anova(lme.1, lme.2)
```

```
##       Model df      AIC      BIC    logLik   Test   L.Ratio p-value
## lme.1     1  6 239.9732 254.9050 -113.9866                         
## lme.2     2  5 238.0905 250.5337 -114.0452 1 vs 2 0.1173278   0.732
```

### Final model

```
lme.3 <- lme(log(Leuco)~Treatment, random=~1|Family, data=Dataset, method="ML", na.action="na.omit")
summary(lme.3)
```

```
## Linear mixed-effects model fit by maximum likelihood
##  Data: Dataset 
##        AIC      BIC    logLik
##   236.1102 246.0647 -114.0551
## 
## Random effects:
##  Formula: ~1 | Family
##         (Intercept)  Residual
## StdDev:   0.2026373 0.8525202
## 
## Fixed effects: log(Leuco) ~ Treatment 
##                 Value Std.Error DF  t-value p-value
## (Intercept)  6.741903 0.1499221 75 44.96939  0.0000
## TreatmentC  -0.450274 0.1850877 75 -2.43276  0.0174
##  Correlation: 
##            (Intr)
## TreatmentC -0.646
## 
## Standardized Within-Group Residuals:
##         Min          Q1         Med          Q3         Max 
## -2.68365025 -0.66537679 -0.01485992  0.55162203  2.43329269 
## 
## Number of Observations: 89
## Number of Groups: 13
```

```
lme.4 <- lme(log(Leuco)~1, random=~1|Family, data=Dataset, method="ML", na.action="na.omit")
anova(lme.3, lme.4)
```

```
##       Model df      AIC      BIC    logLik   Test L.Ratio p-value
## lme.3     1  4 236.1102 246.0648 -114.0551                       
## lme.4     2  3 239.8030 247.2689 -116.9015 1 vs 2 5.69284   0.017
```

## Lymphocytes

### Initial model

```
lme.5 <- lme(log(Lymphoabs)~Sex*Treatment, random=~1|Family, data=Dataset, method="ML", na.action="na.omit")
summary(lme.5)
```

```
## Linear mixed-effects model fit by maximum likelihood
##  Data: Dataset 
##       AIC      BIC    logLik
##   220.395 235.3268 -104.1975
## 
## Random effects:
##  Formula: ~1 | Family
##          (Intercept)  Residual
## StdDev: 4.170502e-05 0.7802192
## 
## Fixed effects: log(Lymphoabs) ~ Sex * Treatment 
##                     Value Std.Error DF  t-value p-value
## (Intercept)      6.209858 0.1742178 73 35.64422  0.0000
## Sexm            -0.086570 0.2409655 73 -0.35926  0.7204
## TreatmentC      -0.612170 0.2322904 73 -2.63536  0.0103
## Sexm:TreatmentC  0.189511 0.3421723 73  0.55385  0.5814
##  Correlation: 
##                 (Intr) Sexm   TrtmnC
## Sexm            -0.723              
## TreatmentC      -0.750  0.542       
## Sexm:TreatmentC  0.509 -0.704 -0.679
## 
## Standardized Within-Group Residuals:
##        Min         Q1        Med         Q3        Max 
## -2.6004788 -0.6617163 -0.1812727  0.6458814  2.0637533 
## 
## Number of Observations: 89
## Number of Groups: 13
```

```
lme.6 <- lme(log(Lymphoabs)~Sex+Treatment, random=~1|Family, data=Dataset, method="ML", na.action="na.omit")
anova(lme.5, lme.6)
```

```
##       Model df      AIC      BIC    logLik   Test   L.Ratio p-value
## lme.5     1  6 220.3949 235.3268 -104.1975                         
## lme.6     2  5 218.7156 231.1587 -104.3578 1 vs 2 0.3206019  0.5712
```

### Final model

```
lme.7 <- lme(log(Lymphoabs)~Treatment, random=~1|Family, data=Dataset, method="ML", na.action="na.omit")
summary(lme.7)
```

```
## Linear mixed-effects model fit by maximum likelihood
##  Data: Dataset 
##        AIC      BIC    logLik
##   216.7175 226.6721 -104.3588
## 
## Random effects:
##  Formula: ~1 | Family
##         (Intercept)  Residual
## StdDev: 4.89125e-05 0.7816343
## 
## Fixed effects: log(Lymphoabs) ~ Treatment 
##                 Value Std.Error DF  t-value p-value
## (Intercept)  6.164606 0.1191825 75 51.72406  0.0000
## TreatmentC  -0.525741 0.1676106 75 -3.13668  0.0024
##  Correlation: 
##            (Intr)
## TreatmentC -0.711
## 
## Standardized Within-Group Residuals:
##        Min         Q1        Med         Q3        Max 
## -2.6486310 -0.7131980 -0.1604787  0.6843631  2.1179116 
## 
## Number of Observations: 89
## Number of Groups: 13
```

```
lme.8 <- lme(log(Lymphoabs)~1, random=~1|Family, data=Dataset, method="ML", na.action="na.omit")
anova(lme.7, lme.8)
```

```
##       Model df      AIC      BIC    logLik   Test L.Ratio p-value
## lme.7     1  4 216.7175 226.6721 -104.3588                       
## lme.8     2  3 224.2291 231.6950 -109.1146 1 vs 2 9.51162   0.002
```

## Neutrophils

### Initial model

```
lme.9 <- lme(log(Neutroabs)~Sex*Treatment, random=~1|Family, data=Dataset, method="ML", na.action="na.omit")
summary(lme.9)
```

```
## Linear mixed-effects model fit by maximum likelihood
##  Data: Dataset 
##        AIC      BIC    logLik
##   294.0196 308.9514 -141.0098
## 
## Random effects:
##  Formula: ~1 | Family
##         (Intercept) Residual
## StdDev:   0.4641852 1.121711
## 
## Fixed effects: log(Neutroabs) ~ Sex * Treatment 
##                     Value Std.Error DF   t-value p-value
## (Intercept)      5.662723 0.2984605 73 18.973108  0.0000
## Sexm            -0.055362 0.3589596 73 -0.154230  0.8779
## TreatmentC      -0.435597 0.3498067 73 -1.245250  0.2170
## Sexm:TreatmentC  0.039538 0.5256741 73  0.075214  0.9402
##  Correlation: 
##                 (Intr) Sexm   TrtmnC
## Sexm            -0.618              
## TreatmentC      -0.663  0.559       
## Sexm:TreatmentC  0.443 -0.719 -0.696
## 
## Standardized Within-Group Residuals:
##        Min         Q1        Med         Q3        Max 
## -2.2143208 -0.6196089  0.1255582  0.6107049  2.7630146 
## 
## Number of Observations: 89
## Number of Groups: 13
```

```
lme.10 <- lme(log(Neutroabs)~Sex+Treatment, random=~1|Family, data=Dataset, method="ML", na.action="na.omit")
anova(lme.9, lme.10)
```

```
##        Model df      AIC      BIC    logLik   Test     L.Ratio p-value
## lme.9      1  6 294.0196 308.9514 -141.0098                           
## lme.10     2  5 292.0255 304.4687 -141.0128 1 vs 2 0.005915996  0.9387
```

### Final model

```
lme.11 <- lme(log(Neutroabs)~Treatment, random=~1|Family, data=Dataset, method="ML", na.action="na.omit")
summary(lme.11)
```

```
## Linear mixed-effects model fit by maximum likelihood
##  Data: Dataset 
##        AIC      BIC    logLik
##   290.0472 300.0017 -141.0236
## 
## Random effects:
##  Formula: ~1 | Family
##         (Intercept) Residual
## StdDev:   0.4659088 1.121593
## 
## Fixed effects: log(Neutroabs) ~ Treatment 
##                 Value Std.Error DF   t-value p-value
## (Intercept)  5.633805 0.2321537 75 24.267567  0.0000
## TreatmentC  -0.412622 0.2464706 75 -1.674124  0.0983
##  Correlation: 
##            (Intr)
## TreatmentC -0.567
## 
## Standardized Within-Group Residuals:
##        Min         Q1        Med         Q3        Max 
## -2.2349770 -0.6321388  0.1051478  0.6134756  2.7458693 
## 
## Number of Observations: 89
## Number of Groups: 13
```

```
lme.12 <- lme(log(Neutroabs)~1, random=~1|Family, data=Dataset, method="ML", na.action="na.omit")
anova(lme.11, lme.12)
```

```
##        Model df      AIC      BIC    logLik   Test  L.Ratio p-value
## lme.11     1  4 290.0472 300.0017 -141.0236                        
## lme.12     2  3 290.8138 298.2797 -142.4069 1 vs 2 2.766587  0.0963
```

## Monocytes

### Initial model

```
lme.13 <- lme(log(Monoabs+1)~Sex*Treatment, random=~1|Family, data=Dataset, method="ML", na.action="na.omit")
summary(lme.13)
```

```
## Linear mixed-effects model fit by maximum likelihood
##  Data: Dataset 
##        AIC      BIC    logLik
##   349.6558 364.5877 -168.8279
## 
## Random effects:
##  Formula: ~1 | Family
##         (Intercept) Residual
## StdDev:   0.6969617 1.522286
## 
## Fixed effects: log(Monoabs + 1) ~ Sex * Treatment 
##                      Value Std.Error DF   t-value p-value
## (Intercept)      2.5885715 0.4158837 73  6.224268  0.0000
## Sexm            -0.2743932 0.4883495 73 -0.561879  0.5759
## TreatmentC      -0.9795983 0.4770604 73 -2.053405  0.0436
## Sexm:TreatmentC  1.1474267 0.7172338 73  1.599795  0.1140
##  Correlation: 
##                 (Intr) Sexm   TrtmnC
## Sexm            -0.604              
## TreatmentC      -0.649  0.560       
## Sexm:TreatmentC  0.433 -0.719 -0.698
## 
## Standardized Within-Group Residuals:
##         Min          Q1         Med          Q3         Max 
## -1.57511275 -0.84528764  0.02067841  0.67525880  2.44533976 
## 
## Number of Observations: 89
## Number of Groups: 13
```

```
lme.14 <- lme(log(Monoabs+1)~Sex+Treatment, random=~1|Family, data=Dataset, method="ML", na.action="na.omit")
anova(lme.13, lme.14)
```

```
##        Model df      AIC      BIC    logLik   Test  L.Ratio p-value
## lme.13     1  6 349.6558 364.5877 -168.8279                        
## lme.14     2  5 350.2412 362.6843 -170.1206 1 vs 2 2.585307  0.1079
```

### Final model

```
lme.15 <- lme(log(Monoabs+1)~Treatment, random=~1|Family, data=Dataset, method="ML", na.action="na.omit")
summary(lme.15)
```

```
## Linear mixed-effects model fit by maximum likelihood
##  Data: Dataset 
##        AIC      BIC    logLik
##   348.9663 358.9209 -170.4832
## 
## Random effects:
##  Formula: ~1 | Family
##         (Intercept) Residual
## StdDev:   0.7743317 1.539607
## 
## Fixed effects: log(Monoabs + 1) ~ Treatment 
##                  Value Std.Error DF   t-value p-value
## (Intercept)  2.4627019 0.3450765 75  7.136684  0.0000
## TreatmentC  -0.4777502 0.3400109 75 -1.405103  0.1641
##  Correlation: 
##            (Intr)
## TreatmentC -0.53 
## 
## Standardized Within-Group Residuals:
##         Min          Q1         Med          Q3         Max 
## -1.56101758 -0.91478101  0.08931124  0.71637011  2.31946272 
## 
## Number of Observations: 89
## Number of Groups: 13
```

```
lme.16 <- lme(log(Monoabs+1)~1, random=~1|Family, data=Dataset, method="ML", na.action="na.omit")
anova(lme.15, lme.16)
```

```
##        Model df      AIC      BIC    logLik   Test  L.Ratio p-value
## lme.15     1  4 348.9663 358.9209 -170.4832                        
## lme.16     2  3 348.9634 356.4293 -171.4817 1 vs 2 1.997051  0.1576
```

## Proportion neutrophils:lymphocytes

### Initial model

```
lme.17 <- lme(log(NeutroToLympho)~Sex*Treatment, random=~1|Family, data=Dataset, method="ML", na.action="na.omit")
summary(lme.17)
```

```
## Linear mixed-effects model fit by maximum likelihood
##  Data: Dataset 
##        AIC      BIC    logLik
##   230.0744 245.0062 -109.0372
## 
## Random effects:
##  Formula: ~1 | Family
##         (Intercept)  Residual
## StdDev:   0.3433999 0.7797794
## 
## Fixed effects: log(NeutroToLympho) ~ Sex * Treatment 
##                      Value Std.Error DF    t-value p-value
## (Intercept)     -0.5320367 0.2108055 73 -2.5238271  0.0138
## Sexm             0.0021940 0.2499201 73  0.0087789  0.9930
## TreatmentC       0.1381549 0.2439081 73  0.5664221  0.5728
## Sexm:TreatmentC -0.1396571 0.3666446 73 -0.3809059  0.7044
##  Correlation: 
##                 (Intr) Sexm   TrtmnC
## Sexm            -0.610              
## TreatmentC      -0.654  0.560       
## Sexm:TreatmentC  0.437 -0.719 -0.697
## 
## Standardized Within-Group Residuals:
##         Min          Q1         Med          Q3         Max 
## -1.85835961 -0.74427203 -0.02734295  0.74575160  2.07771876 
## 
## Number of Observations: 89
## Number of Groups: 13
```

```
lme.18 <- lme(log(NeutroToLympho)~Sex+Treatment, random=~1|Family, data=Dataset, method="ML", na.action="na.omit")
anova(lme.17, lme.18)
```

```
##        Model df      AIC      BIC    logLik   Test   L.Ratio p-value
## lme.17     1  6 230.0744 245.0062 -109.0372                         
## lme.18     2  5 228.2262 240.6694 -109.1131 1 vs 2 0.1517745  0.6968
```

### Final model

```
lme.19 <- lme(log(NeutroToLympho)~Treatment, random=~1|Family, data=Dataset, method="ML", na.action="na.omit")
summary(lme.19)
```

```
## Linear mixed-effects model fit by maximum likelihood
##  Data: Dataset 
##        AIC      BIC    logLik
##   226.3782 236.3327 -109.1891
## 
## Random effects:
##  Formula: ~1 | Family
##         (Intercept)  Residual
## StdDev:   0.3448096 0.7809662
## 
## Fixed effects: log(NeutroToLympho) ~ Treatment 
##                  Value Std.Error DF   t-value p-value
## (Intercept) -0.5314969 0.1655607 75 -3.210285  0.0020
## TreatmentC   0.0813494 0.1718848 75  0.473279  0.6374
##  Correlation: 
##            (Intr)
## TreatmentC -0.556
## 
## Standardized Within-Group Residuals:
##        Min         Q1        Med         Q3        Max 
## -1.8060787 -0.6709673 -0.0355774  0.7808315  2.1600303 
## 
## Number of Observations: 89
## Number of Groups: 13
```

```
lme.20 <- lme(log(NeutroToLympho)~1, random=~1|Family, data=Dataset, method="ML", na.action="na.omit")
anova(lme.19, lme.20)
```

```
##        Model df      AIC      BIC    logLik   Test  L.Ratio p-value
## lme.19     1  4 226.3782 236.3327 -109.1891                        
## lme.20     2  3 224.6002 232.0661 -109.3001 1 vs 2 0.222015  0.6375
```
